# Neuroinflammatory Markers in Former Athletes with Repetitive Head Impacts: Associations with Cognitive Function and White Matter Hyperintensity Volume

**DOI:** 10.1002/alz70856_107558

**Published:** 2026-01-09

**Authors:** Lian Lopes Troncoso, Chloe Anastassiadis, Simrika Thapa, Nusrat Sadia, Mozhgan Khodadadi, Robin Green, Charles Tator, Carmela Tartaglia

**Affiliations:** ^1^ Canadian Concussion Centre, Toronto Western Hospital, University Health Network, Toronto, ON, Canada; ^2^ Tanz Centre for Research in Neurodegenerative Diseases, University of Toronto, Toronto, ON, Canada; ^3^ Department of Surgery, University of Toronto, Toronto, ON, Canada; ^4^ Krembil Brain Institute, University Health Network (UHN), Toronto, ON, Canada

## Abstract

**Background:**

Repetitive head impacts (RHI) is a risk factor for chronic traumatic encephalopathy (CTE), a neurodegenerative disease that includes white matter abnormalities and neuroinflammation. This study examined associations between inflammatory markers, neurodegeneration, white matter integrity, and cognitive performance in former athletes with RHI.

**Method:**

Neuroinflammatory markers were evaluated in the CSF of 16 individuals with RHI (100% male, mean age = 60.9±10.56) and 6 healthy controls (HC) (50% male, mean age = 57±10.56). Using Proximity Extension Assay (PEA), 737 inflammatory markers were quantified in CSF. Neurofilament Light Chain (NfL) was measured using Single Molecule Array (SIMOA), white matter hyperintensity (WMH) volume was assessed on MRI FLAIR, and cognitive performance was evaluated using composite scores for executive function, memory, and mood/behavior (calculated as the mean z‐score of Anxiety, Depression, Mania clinical scales, and Aggression treatment consideration scales from the Personality Assessment Inventory). Mann‐Whitney U Test was used to compare inflammatory markers between RHI and HC. Pearson's correlation was used to assess relationships between inflammatory markers and NfL, while linear regression analyzed associations with cognitive function and WMH volume. Functional enrichment analysis was used to identify associated biological processes.

**Result:**

Significant relationships were found between inflammatory markers and cognitive performance. Memory function was associated with 22 markers (e.g., DPP7, TNFRSF25, WAS), while 24 were linked to executive function (e.g., IL10, CCL11, NUMB). Functional enrichment analysis highlighted tripeptidyl‐peptidase activity (GO:0008240, p_adj <0.001), apoptosis regulation (GO:0042981, p_adj <0.01), cytokine receptor binding (GO:0005126, p_adj < 0.01), and JAK‐STAT signaling (KEGG:04630, p_adj < 0.01). Twenty‐three inflammatory markers were associated with mood/behavior scores (e.g., IL10, CCL7, ITGA11, PTX3), with enrichment in cytokine activity (GO:0005125, p_adj <0.001) and eosinophil chemotaxis (GO:0048245, p_adj < 0.01). DTD1 (*p* < 0.0001, FDR *p* =  0.0002) and LY75 (*p* = 0.0001, FDR *p* <0.05) showed significant relationships with WMH volume. No significant differences in inflammatory marker levels were found between RHI and HC after FDR correction, and none of the 41 markers initially linked to NfL remained significant.

**Conclusion:**

These exploratory findings suggest inflammatory markers may contribute to cognitive and imaging changes in RHI. Replication in larger cohorts is needed.

